# Differential processing of thalamic information via distinct striatal interneuron circuits

**DOI:** 10.1038/ncomms15860

**Published:** 2017-06-12

**Authors:** Maxime Assous, Jaime Kaminer, Fulva Shah, Arpan Garg, Tibor Koós, James M. Tepper

**Affiliations:** 1 Center for Molecular and Behavioral Neuroscience, Rutgers University, Newark, New Jersey 07102, USA

## Abstract

Recent discoveries of striatal GABAergic interneurons require a new conceptualization of the organization of intrastriatal circuitry and their cortical and thalamic inputs. We investigated thalamic inputs to the two populations of striatal neuropeptide Y (NPY) interneurons, plateau low threshold spike (PLTS) and NPY-neurogliaform (NGF) cells. Optogenetic activation of parafascicular inputs evokes suprathreshold monosynaptic glutamatergic excitation in NGF interneurons and a disynaptic, nicotinic excitation through cholinergic interneurons. In contrast, the predominant response of PLTS interneurons is a disynaptic inhibition dependent on thalamic activation of striatal tyrosine hydroxylase interneurons (THINs). In contrast, THINs do not innervate NGF or fast spiking interneurons, showing significant specificity in THINs outputs. Chemospecific ablation of THINs impairs prepulse inhibition of the acoustic startle response suggesting an important behavioural role of this disynaptic pathway. Our findings demonstrate that the impact of the parafascicular nucleus on striatal activity and some related behaviour critically depend on synaptic interactions within interneuronal circuits.

## Abstract (web summary)

The responses of striatal GABAergic interneurons to thalamic inputs are not well characterised. Here, the authors demonstrate that complex intrastriatal circuitry is responsible for thalamic-evoked monosynaptic and disynaptic excitation in NPY-NGF interneurons but a disynaptic inhibition in the NPY-PLTS.

The basal ganglia (BG) are a group of subcortical brain nuclei essential for the control of movement and a variety of other functions^1^,^2^. The striatum is the main input nucleus and receives excitatory afferents from the cortex and the thalamus^3^,^4^,^5^.

The thalamostriatal system is thought to be critical in mediating BG responses to attention-related stimuli, and may be engaged in behavioural switching and reinforcement functions^6^,^7^,^8^,^9^. Although this system originates from several thalamic nuclei, the centromedian/parafascicular complex (CM/Pf), or the parafascicular nucleus (PfN) in rodents, is a principal source of thalamostriatal projections^5^,^10^. Most neurophysiological studies of thalamostriatal afferents have focused on their connections with spiny projection neurons (SPNs)^10^,^11^. PfN afferents also synapse onto cholinergic interneurons (CINs) and several subtypes of striatal GABAergic interneurons^12^,^13^,^14^,^15^.

Striatal interneurons play crucial roles in striatal functioning. While CINs have long been known to be essential for normal functioning of the striatum^16^,^17^,^18^,^19^, GABAergic interneurons, including parvalbumin (PV)-expressing fast-spiking interneurons (FSIs) and tyrosine hydroxylase (TH) and Neuropeptide Y (NPY)-expressing interneurons, exert powerful inhibitory effects on SPNs^20^,^21^,^22^,^23^,^24^,^25^. FSI, PLTS and TH interneurons (THINs) receive powerful excitation from cortex^20^,^21^,^22^,^26^,^27^ resulting in feed-forward inhibition that regulates spike timing in SPNs^23^.

Although it is well established that CINs receive a strong thalamic input^12^,^13^,^15^,^28^, thalamic inputs to specific striatal GABAergic interneurons are less well understood. In non-human primates, most striatal somatostatin (SOM)- and CR-expressing interneurons receive asymmetric synapses from the CM/Pf^15^. In rodents, FSIs receive only sparse PfN inputs^14^ while more recent electrophysiological studies have reported strong excitation of PV interneurons when stimulating the whole thalamus^29^. Interestingly, some authors have been unsuccessful in demonstrating direct PfN inputs to NPY-expressing neurons in rats^30^.

There are two electrophysiologically and morphologically distinct subtypes of striatal NPY interneuron. The PLTS interneurons co-expresses SOM and nitric oxide (NO) synthase while the neurogliaform (NGF) interneurons expresses neither^22^. Regulation of PLTS activity is likely important for striatal plasticity as it has been recently demonstrated that activation of PLTS interneurons produces a postsynaptic NO-mediated long-term depression^31^. On the other hand, the NGF interneuron is the intrastriatal source of powerful, slow GABA_A_ inhibition of SPNs^19^,^22^.

We use optogenetics coupled with *ex vivo* whole-cell recordings to investigate the thalamic input to the two populations of striatal GABAergic NPY interneurons. This is achieved with viral transduction of the PfN with channelrhodopsin2 coupled to a yellow fluorescent protein reporter (ChR2-YFP) under control of a CAM kinase 2 promotor in a BAC transgenic NPY-GFP mouse. Our results reveal that activation of the PfN thalamostriatal pathway evokes very different and highly selective responses in the two NPY interneuron subtypes.

## Results

### Distinctions between the two NPY interneurons populations

**Figure 1:**
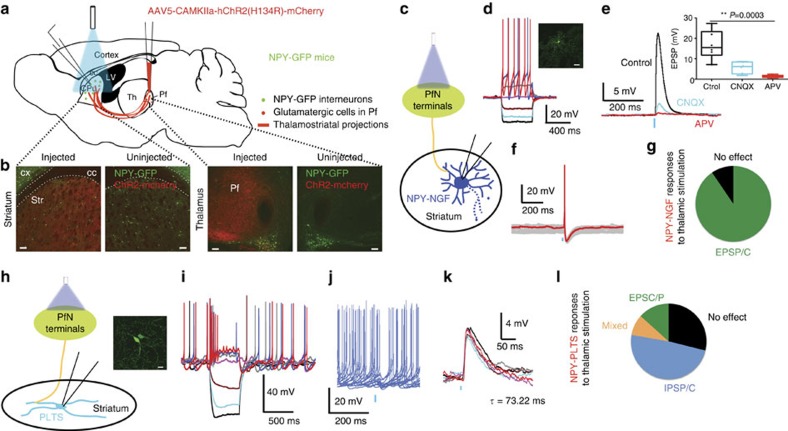
Transduction field in thalamus and input to NPY interneurons in the striatum. (**a**) Schematic illustration of the experimental preparation. (**b**) Representative low-magnification photomicrographs of NPY-GFP cells (green) and ChR2-mcherry (red) transduced PfN axons in striatum (left) and cell bodies in thalamus (right). Scale bar, 100 μM. cc, corpus callosum; LV, lateral ventricle; PfN, parafascicular nucleus. (**c**) Recordings of NGF interneurons were obtained while stimulating PfN terminals in striatum with 2 ms blue light pulses. (**d**) Responses of a typical NGF interneuron to injected current pulses. Note the very large afterhyperpolarization (AHP). Scale bar, 30 μM (**e**,**f**) Current-clamp recordings of an NGF interneuron. Optogenetic stimulation of PfN terminals evokes a large EPSP that is significantly reduced by the application of glutamate receptor antagonists (CNQX 10 μM, *P*=0.0015 versus control and APV 10 μM, *P*<0.0001 versus control; one-way ANOVA followed by Tukey's multiple comparisons test, *n*=6) (**e**). The excitatory response is sufficient to trigger action potential firing ((**f**), *n*=8/21 recorded NGF). (**g**) More than 90% of NGF interneurons are excited by PfN stimulation. (**h**) Recordings of PLTS interneurons while stimulating PfN terminals in striatum with 2 ms blue light pulses. Scale bar, 10 μM. (**i**) Membrane potential responses of a typical PLTS interneuron to injected current pulses. (**j**) Spontaneously active PLTS interneurons showing the inhibition caused by optogenetic PfN stimulation. (**k**) Example of the subthreshold EPSP seen in a minority of PLTS cells. (**l**) Note the high proportion of no response in PLTS interneurons and that the most frequent response was an IPSC/P (*n*=22/45 for IPSP/C, *n*=13/45 for no response, *n*=4/45 for mixed and *n*=6/45 for EPSP/C). Box plots represent the minimum, maximum interquartile range, the mean and median.

As previously described in striatal slices from NPY-GFP animals (Ibáñez-Sandoval *et al*.^22^), GFP fluorescent somata were composed of two distinct populations of medium-sized neurons. The majority of these exhibited moderately bright fluorescence and could be seen to emit from one to three straight, aspiny primary dendrites that could only be followed for a short distance. These cells, previously identified as NPY-PLTS interneurons^22^,^32^, exhibited high input resistance, a time-dependent I_h_-like sag in response to hyperpolarizing current injections, and a relatively depolarized resting membrane potential. PLTS interneurons usually exhibited spontaneous activity and a prominent low-threshold spike (LTS) in response to depolarizing current injections from rest or as a rebound following the offset of hyperpolarizing current pulses. The most characteristic trait of NPY–PLTS interneurons was a prolonged plateau potential (Fig. 1c) following the termination of depolarizing or hyperpolarizing current injections^22^,^26^,^32^,^33^. A second, smaller population of interneurons exhibited extremely bright fluorescence and issued five to nine primary aspiny dendrites (Fig. 1d). Due to their electrophysiological and morphological homology with cortical and hippocampal GABAergic NPY-expressing interneurons in the hippocampus and cortex^34^,^35^,^36^,^37^ they were identified as NPY–NGF interneurons^22^. NPY-NGF interneurons exhibited a significantly lower input resistance and hyperpolarized membrane potential, were never spontaneously active, and displayed regular, non-accommodating spiking in response to depolarizing current injections, and an absence of plateau depolarizations or low-threshold spikes. NPY–NGF interneurons also exhibited a spike afterhyperpolarization that was notable for its large amplitude (Fig. 1d).

### Innervation of NPY interneurons by the PfN of the thalamus

NPY-positive interneurons were targeted in an NPY-GFP transgenic mouse strain, while PfN axons were activated optogenetically using AAV5-mediated gene transfer of a ChR2(H134R)-m-cherry transgene controlled by a CAMK-II promoter. Efficacy and confinement of the transduction within the PfN was demonstrated anatomically (Fig. 1a,b; Supplementary Fig. 1a–d).

Previous work from many labs has described various aspects of the powerful excitation exerted by intralaminar thalamic nuclei onto SPNs^5^,^10^,^11^,^38^. Those studies showed that thalamostriatal synapses onto SPNs exhibit a high release probability and are depressed by repetitive stimulation^10^,^11^. In our hands, optogenetic stimulation of PfN thalamic terminals elicited large EPSPs in SPNs when recorded in current clamp (*n*=8; 23.31±4 mV, *τ*=10.43±1.08 ms, Supplementary Fig. 2b,c). In voltage clamp a large EPSC (*n*=7; 607.68±11.72 pA, *τ*=5.95±0.29 ms) was induced (Supplementary Fig. 2d,f). Those responses were confirmed to be glutamatergic as they were blocked by bath application of CNQX and APV (respectively AMPA and NMDA receptor antagonists, 10 μM Supplementary Fig. 2b,e).

NGF interneurons responded to optogenetic activation of PfN axons with large amplitude EPSC/Ps (90.5%, *n*=19/21 recorded NGF). The average EPSP amplitude was 13.03±2.21 mV (Fig. 1e; Supplementary Fig. 3c; *τ*=23.1±3.3 ms; *n*=12) and at *V*_h_=−70 mV, the average EPSC amplitude was 219.72±55.4 pA (Supplementary Fig. 3c,d; *τ*=13.8±5.48 ms; *n*=14). This EPSP/C was a mixed AMPA/NMDA monosynaptic response that exhibited latency invariance and was blocked by bath application of selective glutamatergic antagonists (Fig. 1e; CNQX, 10 μM; and APV, 10 μM). The optically evoked EPSP exhibited a 59.6±11.4% reduction after CNQX (paired *t*-test; *P*=0.018) and an 89.9±2.17% reduction after CNQX+APV; *n*=6; paired *t*-test, *P*=0.0003)]. The EPSC exhibited use dependent depression to brief stimulus trains (5 stimuli at 20 Hz; *n*=7; Supplementary Fig. 3f).

In 8 of 21 NGF cells (38.09%), stimulation of PfN axons elicited action potentials (Fig. 1f). Application of bicuculline (10 μM) did not significantly affect synaptic response amplitudes demonstrating that disynaptic GABAergic inputs were not activated (*n*=6; Supplementary Fig. 3g,h).

In marked contrast to NGF interneurons, a significant proportion of PLTS interneurons 13/45 (28.89%) failed to exhibit any response to stimulation of PfN terminals (Fig. 1l; Supplementary Fig. 4m). Of the remainder, the largest fraction (22/45, 48.89%) responded to the same stimuli with large amplitude pure inhibitory responses (Fig. 1j,l). This difference could also be observed when recording neighbouring PLTS and NGF interneurons in the same slice (Supplementary Fig. 5; *n*=4).

A minority of PLTS cells (8.89%, *n*=4/45 recorded cells) exhibited small amplitude excitatory responses isolated by blocking synaptic inhibition (Supplementary Fig. 3k,l) or represented the only observable synaptic response (13.33%, *n*=6/45 of recorded neurons). These EPSP/Cs were relatively small, 5.77±2.06 mV in amplitude (*τ*=73.22±13.65 ms; *n*=3; Fig. 1k; Supplementary Fig. 3b,d; time delay: 7.225±0.1522, ms and time to peak: 10.47±1.016 ms; Supplementary Fig. 6a–c, and 20.32±4.59 pA, *τ*=6.55±0.91 ms at −70 mV; *n*=7; Supplementary Fig. 3c,e) and were blocked by AMPA and NMDA receptor antagonists (*n*=3; Supplementary Fig. 3l). These EPSPs were insufficient to trigger action potentials, depolarizing plateaus potentials or low-threshold spikes despite the high input resistance of PLTS neurons (*n*=9; Fig. 1k; Supplementary Fig. 3b,l). In contrast to EPSCs in NGF interneurons, the EPSC did not exhibit significant use dependent depression (*n*=6, Supplementary Fig. 3j,l).

**Figure 2:**
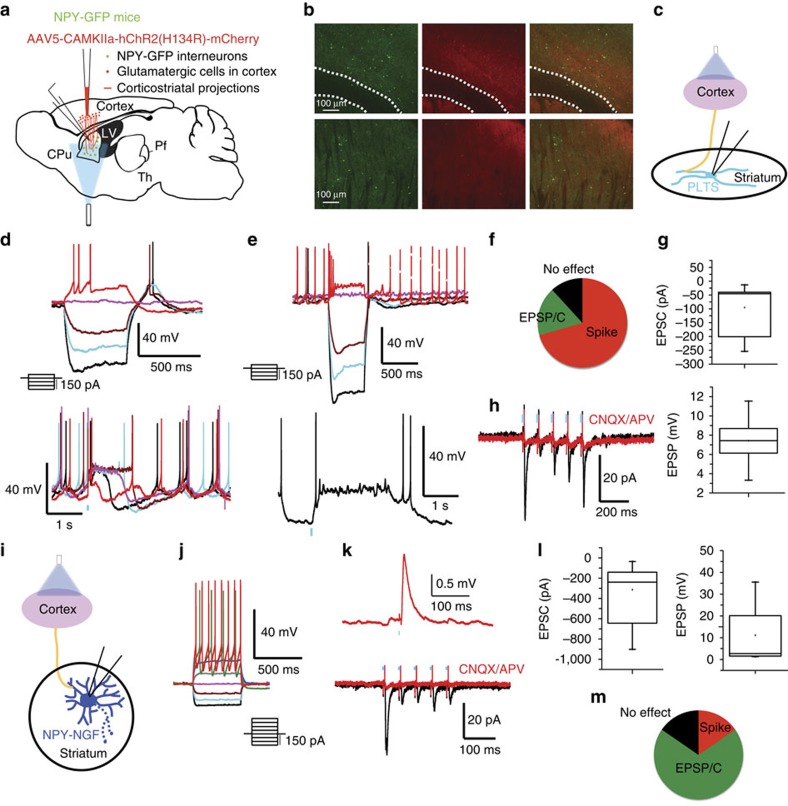
NPY-PLTS and NPY-NGF interneurons respond differentially to optogenetic stimulation of cortex. (**a**) Schematic of the experimental paradigm for optogenetic activation of corticostriatal inputs and recording NPY-expressing interneurons in the striatum. (**b**) Confocal images of the transduction field in the cortex (upper panels) and in the striatum (lower panels). Green channel represents the NPY-GFP interneurons and red channel represents the CAMKII-ChR2-mCherry virus transduction. (**c**–**h**) Response of PLTS to cortical optogenetic stimulation. (upper panels in **d** and **e**) Responses of typical PLTS interneurons to injected current pulses. (**d**,**e**) Optogenetic stimulation of the cortex elicits large depolarization, action potential firing and plateau potentials in NPY-PLTS interneurons. (**f**) Percentage of PLTS cells that responded to stimulation of the cortex. Note that the vast majority of PLTS cells spike after cortical stimulation. (**g**) Box plots representing the amplitude of the EPSP/C evoked by the optogenetic stimulation (*n*=5 and *n*=10, respectively). (**h**) Voltage-clamp recording (Vh=−70 mV) showing that the excitatory response is blocked by CNQX and APV, 10 μM. (**i**–**m**) Response of NGF interneurons to cortical stimulation. (**j**) Responses of typical NGF interneurons to injected current pulses. (**k**) Current-clamp and voltage-clamp recording of an NPY-NGF interneuron showing that optogenetic cortical stimulation provokes EPSP/Cs that are blocked by ionotropic glutamate receptor antagonists. (**l**) Box plots representing the amplitude of the EPSP/C provoked by the optogenetic stimulation (*n*=8 for both EPSP and EPSC). (**m**) Percentage of NPY-NGF cells that responded to optogenetic stimulation of the cortex. Note that although many NGF cells are excited by cortical stimulation only a minority of them spike. Blue bars indicate optical stimulation.

Consistent with results from electrical stimulation^22^, optogenetic cortical stimulation evoked excitatory responses in the majority of PLTS interneurons (*n*=15/17; 88,2%; EPSP/C amplitude: 7.43±1.36 mV and −95.23±30.16 pA; Fig. 2d–h). This was sufficient to trigger action potential firing and plateau potentials in most (70.58%, *n*=12/17) PLTS interneurons. This response was confirmed to be glutamatergic as it was blocked by 10 μM CNQX and 10 μM APV; Fig. 2h; *n*=5). NGF interneurons also received glutamatergic input from cortical optogenetic stimulation (*n*=11/13; EPSP/C amplitude: 11.16±4.48 mV and −313.4±108.67 pA; Fig. 2k). However, in contrast to PLTS interneurons, and similar to results obtained with electrical stimulation^23^, optogenetic cortical stimulation was only able to elicit action potential firing in a minority of NGF interneurons (15.38%; *n*=2/13, Fig. 2m). These results, that the PLTS interneurons are predominantly excited by cortical input whereas the NGF interneurons are predominantly excited by thalamic input, exemplify the need to stop considering the cortex and thalamus together as a sort of unitary source of glutamatergic excitation of striatal neurons.

### PfN inputs induce disynaptic excitation of NGF interneurons

**Figure 3:**
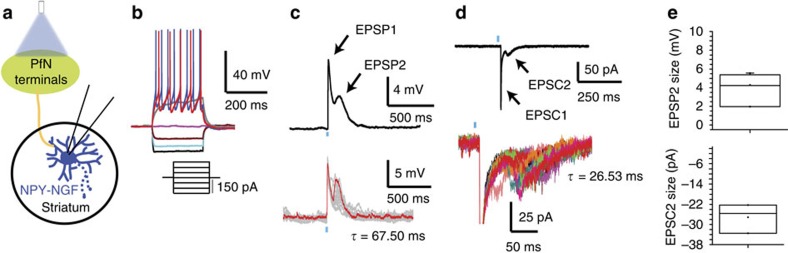
Excitation in NGF interneurons after thalamic stimulation can be biphasic. (**a**) Schematic of experiment (**b**) Membrane potential responses characteristic of a typical NGF interneuron to injected current pulses. (**c**) Optogenetic stimulation of thalamus evoked two kinetically distinct EPSPs. EPSP1 is monosynaptic and shows no latency variability while EPSP2 is later, slower, and shows considerable variability in onset latency. Upper panel: average of 20 sweeps in one neuron. Lower panel: individual traces. (**d**) Voltage clamp (Vh=−70 mV) in the same neuron shows a dual EPSC with the early component (EPSC1) larger and faster than the late component (EPSC2). Upper panel: average of 20 sweeps. Lower panel: individual traces. Note the large variation in the onset latency of EPSC2. (**e**) Summary box plots of the average optogenetic EPSP/C2 (*n*=6). Box plots represent the minimum, maximum interquartile range, the mean and median.

**Figure 4:**
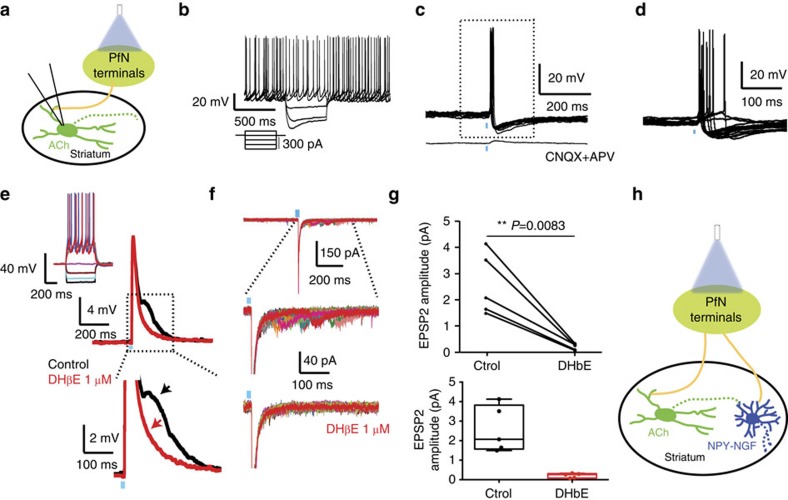
CINs are responsible for the late component of the EPSP/C of NGF interneurons after thalamic stimulation. (**a**) Schematic of the effect of optogenetic stimulation of PfN on CINs. (**b**) Membrane potential responses of a typical spontaneously active CIN to injected current pulses. (**c**) 5 ms light pulse evokes depolarization and spiking. (**d**) Higher-resolution view of the sweeps in **c**. Note the large variability in the latency to spiking following the optical pulse (*n*=4). (**e**) Upper: membrane potential responses of a typical NGF interneuron to injected current pulses. Middle and bottom: optogenetic stimulation of PfN terminals evokes a two component EPSP (black trace, control). The late EPSP is completely blocked by bath application of 1 μM DHßE (red traces) without affecting the first EPSP (*n*=5). (**f**) Voltage-clamp recording (Vh=−70 mV) of a NGF interneuron during optogenetic stimulation of PfN terminals. Higher-resolution sweeps illustrate the variability in the onset latency of the late EPSC. The late EPSC is completely abolished by bath application of DHßE (lower panel). (**g**) Graphs showing the reduction of the EPSP2 size after DHßE (*P*=0.0083 paired *t*-test, *n*=5). (**h**) Summary schematic illustrating the circuitry responsible for early and late synaptic responses. Box plots represent the minimum, maximum interquartile range, the mean and median.

In addition to the monosynaptic glutamatergic effects, optogenetic activation of PfN striatal afferents also activated polysynaptic circuits, giving rise to a long-latency depolarization in NGFs and GABAergic inhibition in PLTS neurons. In addition to the fast EPSP/C, in *n*=6/21 (28.57%) cells, a second, delayed depolarizing synaptic response was also observed (Fig. 3). These late responses (referred to as EPSP/C2) had smaller amplitudes (amplitude EPSP/C2=4.29±0.83 mV and 27.12±3.33 pA at Vh=−70 mV *n*=6) and longer time constants (67.5±11.8 ms for EPSP2 and 26.53±10.75 ms for EPSC2; Fig. 3c–e; *n*=6) than EPSP/C1. The onset latency of EPSP2 varied from trial to trial and cell to cell (Fig. 3d) suggesting involvement of a polysynaptic pathway that likely involves an excitatory striatal interneuron. Previous studies have shown that NGFs receive strong nicotinic excitation from CINs^19^,^39^, which in turn receive excitatory inputs from the thalamus. In additional experiments, we recorded the responses of CINs to stimulation of PfN axons identical to that described for NPY interneurons above that confirmed that action potentials were triggered in CINs that preceded the onset of the delayed EPSP in NGFs. These action potentials showed significant variability in onset latency, consistent with the variable onset latency of EPSC2 (Fig. 4a–c).

Blockade of β2-subunit expressing type II nicotinic receptors with dihydro-β-erythroidine (DHβE, 1 μM) abolished the long-latency response, confirming it as a nicotinic EPSP (Fig. 4e–h; amplitude EPSP2 control: 2.569±0.527 mV vs. amplitude EPSP2 DHβE: 0.2114±0.052 mV, paired *t*-test *P*=0.0083, *n*=5).

Interestingly, the same thalamic activation that elicited action potentials in CINs and downstream nicotinic EPSP/Cs in NGFs failed to trigger any detectable nicotinic synaptic responses in PLTS neurons, demonstrating a differential and highly selective connectivity between CINs and the two types of NPY expressing interneurons.

### PfN inputs induce disynaptic inhibition of PLTS interneurons

**Figure 5:**
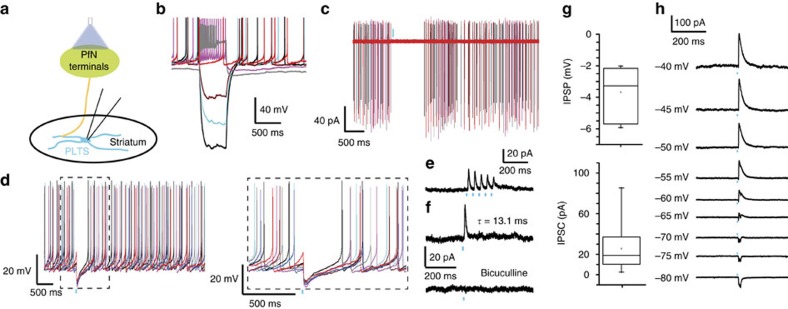
Optogenetic stimulation of the thalamus induces inhibition in most PLTS interneurons. (**a**) Schematic of experiment. (**b**) Responses of a typical PLTS interneuron to injected current pulses. (**c**) Cell-attached recording of same PLTS interneuron showing optogenetic stimulation (blue bar) induced inhibition of spontaneous activity (*n*=4). (**d**) Another spontaneously active PLTS interneuron whose firing is completely inhibited by a 2 ms blue light pulse in current clamp (*n*=14). Lower panel: higher-resolution trace of area shown in dashed box shown in the upper panel. (**e**) PLTS interneuron following short optical train (2 ms at 20 Hz) elicits depressing IPSCs to each stimulus in train. (**f**) Upper panel: single IPSC evoked by 2 ms optical light pulse is blocked completely by 10 μM bicuculline (*n*=8). (**g**) Box plots representing the amplitude of the optogenetic IPSP/C. (**h**) Voltage-clamp recordings at variable holding potentials (from −80 to −40 mV) show that the IPSC reverses close to −70 mV. Box plots represent the minimum, maximum interquartile range, the mean and median.

Next, we investigated the GABAergic inhibition in PLTS neurons evoked by stimulation of PfN terminals. The optogenetically evoked IPSC was 27.7±4.2 pA (*n*=22; *τ*=12.9±0.22 ms; holding potential −45 mV; Fig. 5e–h) and was confirmed to be GABA_A_-mediated by its abolishment by 10 μM bicuculline and a reversal potential close to −70 mV (*n*=8; Fig. 5f,h). Importantly, the inhibition efficiently blocked firing of action potentials in spontaneously active NPY-PLTS interneurons (Fig. 5c,d; *n*=14). That the IPSC results from a disynaptic connection was suggested by the facts that the onset latency and time to peak of the IPSC were significantly longer than for the monosynaptic responses of the other striatal neuron types (Supplementary Fig. 6a–c; onset latency: 9.56±0.31 ms; time to peak: 14.31±0.505 ms; *n*=21, one-way ANOVA, *P*=0.0026 and *F*(3,49)=41.1 for latency and *P*<0.0001 for time to peak). We propose that the IPSP/C results from thalamic excitation of another population of striatal GABAergic interneuron that synapses onto PLTS interneurons (see below). Train stimuli (five 5 ms optical pulses at 20 Hz) elicited time-locked trains of IPSC/Ps (Fig. 5e).

**Figure 6:**
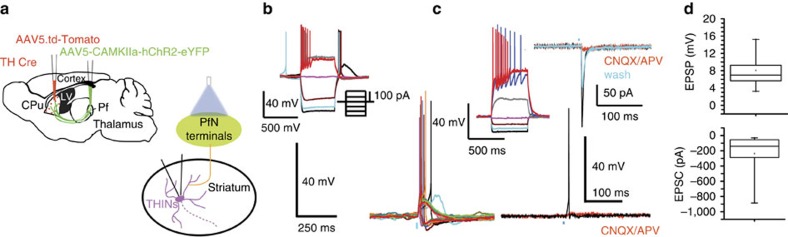
Thalamic input to THINs. (**a**) Schematic of experiments (**b**–**d**). (**b**) Responses of typical type I THIN to injected current pulses. 5 ms optical stimulation of PfN terminals (blue bar) evokes large, often suprathreshold EPSPs (*n*=23, 18/23 were firing action potential). (**c**) Responses of another type I THIN to current pulses. The same cell in voltage clamp responds to same optical stimulation with a large EPSC that is completely abolished by 10 μM CNQX and APV (*n*=9). Lower panel: complete block of EPSP and spiking by 10 μM CNQX and APV (*n*=9). (**d**) Summary box plots of mean EPSP and EPSC amplitudes. Box plots represent the minimum, maximum interquartile range, the mean and median.

**Figure 7:**
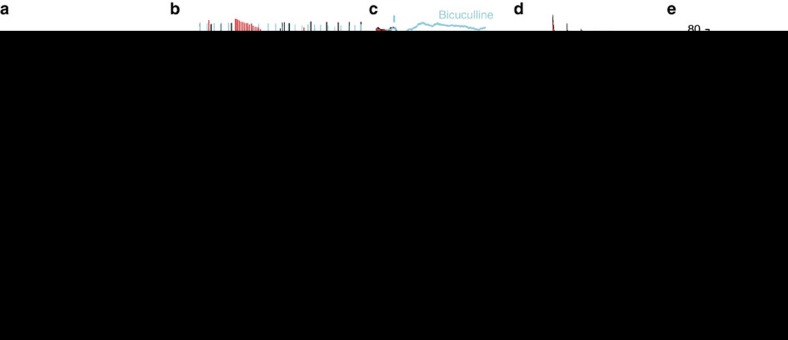
PLTS interneurons receive synaptic input from THINs. (**a**) Schematic of experimental preparation. (**b**) Responses of a typical PLTS interneuron to injected current pulses. (**c**) Optogenetic activation of THINs evokes an IPSP in a PLTS interneuron (*n*=27/32 recorded PLTS) that is blocked by 10 μM bicuculline (*n*=11). (**d**) Brief train (5 2 ms pulses at 20 Hz) evokes short-term depression in the IPSC that is blocked by bicuculline (*n*=9) (Vh=−45 mv). (**e**) Box plots showing mean amplitudes of the IPSC and IPSP. (**f**) Optogenetic stimulation of TH interneurons inhibits spontaneous firing of a PLTS interneuron (*n*=13). Lower panel: higher-resolution traces of the portion of spike train above indicated by the dashed line to show IPSP. (**g**) Upper: after injecting a 50 pA current pulse that induces firing, optogenetic activation of THINs (blue bar) induces a brief pause in firing. Lower: with 100 pA current pulse, the PLTS interneuron enters depolarization block that is relieved by optogenetic activation of THINs. (**h**) Optogenetic inhibition of a spontaneously active PLTS interneuron is reversibly blocked by bicuculline. Box plots represent the minimum, maximum interquartile range, the mean and median.

We hypothesized that part or all of the inhibition may be mediated by THINs, and tested the necessary conditions that (1) PfN inputs activate THINs (Fig. 6) and (2) that THINs inhibit PLTS neurons (Fig. 7). In TH-Cre mice, PfN neurons were transduced with ChR2-eYFP as described above, while THINs were transduced in TH-Cre mice with a floxed td-Tomato reporter for fluorescence-guided recording (Fig. 6a). Optogenetic activation of PfN axons elicited large amplitude EPSP/Cs in all (*n*=23) THINS (Fig. 6b–d; 8.08±0.95 mV, *τ*=43.33±12.9 ms, *n*=4; or a large EPSC (−235.46±60.60 pA, *τ*=3.9±0.4 ms; *n*=19). This excitatory effect was confirmed to be glutamatergic by blockade by (*n*=9; 10 μM CNQX and 10 μM APV). The onset latency and time to peak of the EPSP were consistent with monosynaptic activation (onset latency of 6.32±0.34 ms and time to peak of 13.09±1.66 ms; *n*=19). Most (18/23, 78.26%) THINs responded to thalamic stimulation with firing of action potentials (Fig. 6b,c).

In transgenic *TH-Cre::NPY-GFP* mice injected with a cre-dependent ChR2 virus, optical stimuli triggered action potentials in all THINs that in turn elicited large amplitude IPSP/Cs in PLTS interneurons (Fig. 7; *n*=27/32 recorded PLTS). In spontaneously active PLTS interneurons (*n*=13; Fig. 7f–h), optogenetic activation of THINs produced complete inhibition of firing that was blocked by 10 μM bicuculline (*n*=11). In silent neurons, stimulation resulted in a GABAergic IPSP (−7.66±1.21 mV; *τ*=111.19±59.6 ms; *n*=9, Fig. 7c,e) or IPSC (25.74±3.73 pA; *τ*=16.77±1.53 ms; holding potential −45 mV; *n*=24; Fig. 7d,e) shown to be GABAergic as it was blocked by 10 μM bicuculline (*n*=11; Fig. 7c,d,h).

**Figure 8:**
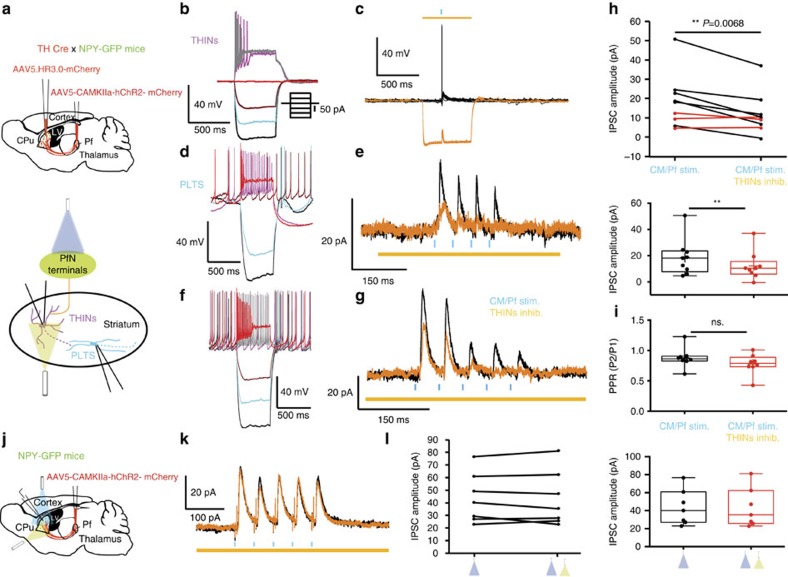
Optogenetic inhibition of THINs greatly decreases or eliminates PfN-evoked IPSCs in PLTS interneurons. (**a**) Schematic representing experimental paradigm for simultaneous activation of PfN and shutdown of THINs. (**b**) Responses of a typical THIN to injected current pulses. (**c**) 500 ms yellow light pulse evokes a strong inhibitory effect on this THIN in current clamp that is sufficient to prevent the THIN from spiking after optogenetic PfN stimulation (*n*=6). (**d**,**f**) Responses of two different typical PLTS interneurons to injected current pulses. (**e**,**g**) The PLTS interneurons exhibit depressing IPSCs to brief optogenetic stimulus trains to PfN terminals (black traces). When PfN stimulation is paired with optogenetic silencing of the THINs by HR3.0, the IPSCs induced by the thalamic stimulation are reduced (orange traces). (**h**) Graphs representing the reduction of the IPSC size after silencing the THINs. Black traces represent average responses in PLTS, where HR3.0 significantly affected the IPSC amplitude, red traces represent average responses in PLTS where HR3.0 failed to do so (*n*=9; *P*=0.0068, paired-sample *t*-test). Lower graph: box plots representing the minimum, maximum interquartile range, the mean and median of the same experiment as in the upper graph. (**i**) Box plots representing the paired-pulse ratio (PPR) of this experiment, where the amplitude of the second IPSC is divided by the amplitude of the first one. No significant differences have been measured after silencing the THINs with HR3.0 (paired *t*-test *P*=0.3239, *n*=8). (**j**) Schematic representing experimental paradigm for controlling the effect of yellow light on the IPSC size. In NPY-GFP mice, we measure the IPSC amplitude provoked by optogenetic stimulation of the PfN in the presence or absence of yellow light. (**k**) The PLTS interneurons exhibit IPSCs to brief optogenetic stimulus trains to PfN terminals (black traces). When PfN stimulation is paired with yellow light, the IPSCs induced by the thalamic stimulation are not affected (orange traces, *n*=7). (**l**) Line graphs (left) and box plots (right) quantifying the IPSC size in the presence or absence of yellow light. No significant effect was measured (*n*=7, *P*=0.7351 paired *t*-test).

While these results confirm the hypothesis that the disynaptic inhibition of PLTS neurons by thalamic activation is at least in part mediated by THINs, the possibility remains that the contribution of THINs may account for only part of the disynaptic inhibition. To investigate the specific contribution of THINs, we examined the effect of optogenetic inhibition of THINs on the synaptic response of PLTS neurons to simultaneous activation of PfN inputs. THINs were transduced with a cre-dependent HALO3.0-mCherry in double transgenic *TH-Cre::NPY-GFP* mice in which the PfN axons were targeted to express ChR2-eYFP (Fig. 8a). Recording from THINs demonstrated that activation of HALO3.0 with yellow light elicited large amplitude hyperpolarizing responses preventing them from firing an action potential after thalamic stimulation (*n*=6; Fig. 8b,c). Synaptic responses in PLTS interneurons to PfN activation were recorded over successive trials in which PfN stimulation trains were paired on alternating trials with optogenetic inhibition of THINs. Yellow light pulses were initiated 150 ms before the onset of PfN stimulation and lasted for 500 ms. As shown in Fig. 8d–i, optogenetic inhibition of THINs significantly reduced the amplitude of the IPSC/P in PLTS neurons (*n*=9) in 66.67% of the PLTS interneurons tested (*n*=6/9). The decrease in the IPSC amplitude ranged from 18.6% to 73.7% (IPSC mean amplitude:18.64±4.64 for PfN stimulation only versus 12.2±3.58 for PfN stimulation and THINs inhibition *P*=0.0068; *n*=9). With optical train stimulation of the PfN terminals (five pulses, 20 Hz), the IPSC in PLTS interneurons was completely abolished to the latter stimuli in the train (Fig. 8e,g; *n*=8). The paired-pulse ratio was the same in both conditions (Fig. 8i; *P*=0.3239). The effect of yellow light alone was controlled for in HALO3.0 negative mice where no significant difference was measured (Fig. 8j–l, *n*=7, *P*=0.7351, paired *t*-test). These results confirm that at least a significant fraction of the disynaptic inhibition originates from THINs.

### Intrastriatal interneuronal synaptic connectivity

Our findings demonstrate a high degree of selectivity and cell-type specificity in the connection of several striatal interneuron subtypes with respect to their thalamic innervation and connections to other interneurons. The observation that the selectivity of innervation by the PfN is maintained through two downstream disynaptic circuits suggests that intrastriatal interneuronal interactions are coordinated in a way that ensures a net opposite effect on two distinct inhibitory mechanisms in the neostriatum.

In contrast to PLTS interneurons NGF interneurons exhibited a purely glutamatergic excitation to activation of PfN axons. Since most THINs are spontaneously active *in vitro*^21^, we hypothesized that these interneurons form highly selective, cell-type-specific connections with the NPY interneurons. To test this, we recorded NGF neurons while optogenetically stimulating THINs in the double transgenic preparation described above.

**Figure 9:**
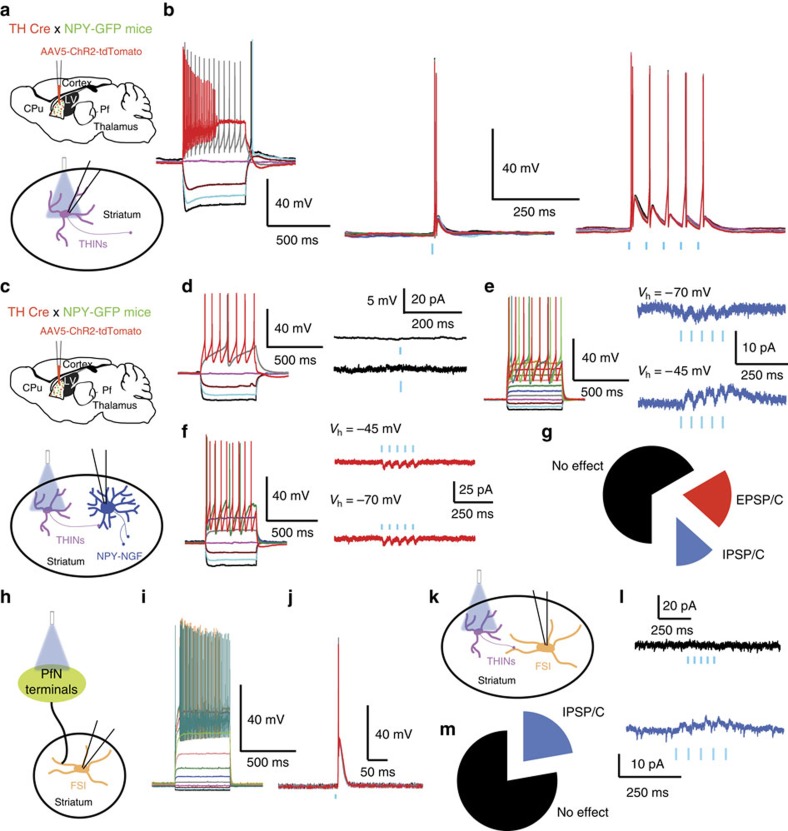
THINs innervation of NPY-NGF and FSI. (**a**) Schematic of the experimental paradigm for recording ChR2-expressing THINs in double transgenic *TH Cre::NPY-GFP* mice. (**b**) Responses of a typical THIN to injected current pulses. Middle and right panels show that brief blue light pulses evoke action potential firing in THINs. (**c**) Schematic of the experimental paradigm for recording NPY-NGF interneurons and optogenetically stimulating THINs. (**d**–**f**). Responses of typical NGF to injected current pulses. (**d**,**g**) Most NGF interneurons do not receive any input from THINs (upper panel: current clamp, lower panel: voltage clamp; Vh=−45 mV). (**e**,**g**). A very small proportion of NGF interneurons respond with a tiny (<4 pA) IPSC after optogenetic THINs stimulation. (**f**,**g**) A very small proportion of NGF receive an excitatory response after optogenetic THINs stimulation. (**h**) Schematic of the experimental paradigm for in recording FSI and activating PfN axons optogenetically. (**i**) Responses of typical FSIs to injected current pulses. (**j**) Optogenetic stimulation of the PfN induces a large excitatory response in recorded FSI that can induce action potential firing. (**k**) Schematic of the experimental paradigm for recording striatal in TH Cre mice and activating THINs optogenetically. (**l**, black trace) Most FSI do not respond to activation of THINS. THINs stimulation (voltage-clamp recording Vh=−45 mV; *n*=7/9, **m**). (**l**, blue trace) Example of an FSI receiving a very small inhibitory response after THINs optogenetic stimulation (voltage-clamp recording Vh=−45 mV; *n*=2/9, **m**).

In most cases (66.7%; *n*=10/15 of NGF interneurons, Fig. 9c,d,g) there were no synaptic responses in NGFs to optogenetic activation of THINs. In a small subset of cells (20%, 3/15) very small amplitude inward currents were triggered by the optical pulse (Fig. 9f,g). These responses could not be blocked by GABA_A_, AMPA or NMDA receptor antagonists, and were insensitive to the holding potential (Fig. 9f; *n*=3). These may represent weak electrotonic or ephaptic coupling and/or ChR2-induced local field potentials. In the remaining two neurons (13.3%), THIN activation led to extremely small (∼4 pA) very slow outward currents of undetermined origin that were not further investigated (Fig. 9e). Thus, the PfN evoked disynaptic inhibition of NPY cells via THINs activation is restricted to the PLTS interneurons.

We also tested potential connections between THINs and FSIs. Unlabelled FSI were recorded in *TH Cre::NPY-GFP* mice, identified by their electrophysiological properties^23^,^32^ (Fig. 9i). Thalamic stimulation elicited suprathreshold monosynaptic excitation in all FSIs (*n*=10; Fig. 9i,j).

Optogenetic activation of THINs elicited only very small inhibitory responses (<5 pA) in a minority of the FSIs tested (*n*=2/9, Fig. 9l, blue trace), while the remainder showed no response (*n*=7/9; Fig. 9l, black trace).

These connections and lack thereof are summarized graphically in Supplementary Fig. 7.

### THINs lesions induces impairment in prepulse inhibition

In the previous section, we demonstrated the importance of THINs in the disynaptic pathway leading to the inhibition of the PLTS interneurons after optogenetic stimulation of the parafascicular nucleus of the thalamus. To investigate the behavioural role of this pathway, we measured the habituation and prepulse inhibition (PPI) of the acoustic startle reflex in mice in which THINs were selectively ablated by intrastriatal injection of a Cre-dependent diphtheria neurotoxin in TH-Cre mice.

**Figure 10:**
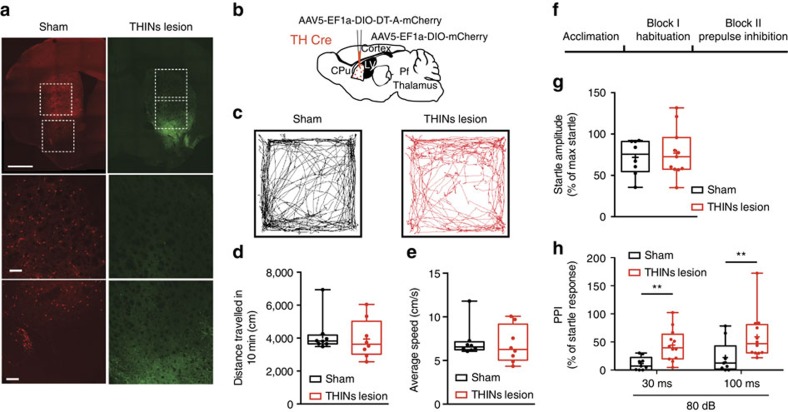
Lesion of THINs induces impairment of the prepulse inhibition to an acoustic startle reflex. (**a**) Confocal microscope pictures representing the Sham-injected animals (red, left panel, AAV5-EF1a-DIO-mCherry) and the TH-lesioned mice (green, right panels, AAV5-EF1a-DIO-DT-A-mCherry + AAV5-EF1a-DIO-ChR2-eYFP). In some cases Sham animals were injected with a different Cre-dependent-mCherry virus (see Methods). Note the drastic lesion of THINs with only a few cells remaining in the ventral striatum (right panels, green). scale bar, 1 mm (upper panels); scale bar, 150 μm (lower two panels). (**b**) Schematic illustrating the experiment paradigm. (**c**) Open-field tracking of a representative Sham animal (left, black) and TH-lesioned animal (right, red). (**d**,**e**) Box blox quantifying respectively the distance travelled and the average speed during 10 min in the open field showing no significant differences between the two groups (respectively *P*=0.6683 and *P*=0.7204; unpaired *t*-test, *n*=8 animals per group). (**f**) Schematic illustrating the habituation and prepulse inhibition paradigm. (**g**) Box blox quantifying the habituation score showing no significant differences between the two groups (*P*=0.6732, unpaired *t*-test *n*=8 Sham and *n*=11 TH-lesioned mice). (**h**) Box blox quantifying the prepulse inhibition of an acoustic startle stimulus after an 80 dB prepulse at two different interstimulus intervals (30 and 100 ms). The quantification shows a significant impairment of the prepulse inhibition in those conditions in TH-lesioned mice (*P*=0.0043 for the 30 ms interval and *P*=0.0357 for the 100 ms interval unpaired *t*-test, *n*=8 Sham and *n*=11 TH-lesioned).

PPI has been linked to the thalamostriatal pathways both in humans^40^,^41^ and specifically to the PfN in rodents^42^. Impairment of PPI results from experimental lesions of striatum^43^,^44^ or thalamus^45^ and are affected in humans with schizophrenia and Huntingtons's disease^46^,^47^. We used TH-Cre mice in which we injected an AAV-5 encoding Cre-dependent diphtheria toxin fragment A (DT-A) (or Cre-dependent m-Cherry virus for the Sham group) into the striatum to selectively lesion TH interneurons, Fig. 10a. Four weeks after injection, we measured spontaneous locomotor activity of these in an open-field arena. There was no difference between the THINs lesion and sham groups either in terms of distance travelled (Fig. 10c,d; 4,200±400 cm for sham versus 3,946±420 cm for THINs lesion *P*=0.67; *n*=8 per group) or average speed (Fig. 10e; 7.2±0.67 cm s^−1^ for sham versus 6.83±0.75 for THINs lesion *P*=0.72; *n*=8 per group), suggesting that lesioning the THINs does not induce any gross motor deficits.

Then we measured habituation and PPI of an acoustic startle stimulus. There was no significant difference in the habituation ratio (% of last five startle trials by the maximal startle value obtained in the first three trials) between the sham group and the THINs lesion group (Fig. 10f,g; 71.8±7.5% for the sham group versus 76.98±8.8% for the THINs group; *P*=0.6732; *n*=8 sham and *n*=11 THINs lesion). However in marked contrast, there was a significant decrease in the PPI (at 80 dB), at both interstimulus intervals tested (Fig. 10h; 30 ms: 11.43±3.96% for sham versus 44.41±8.26% for THINs lesion; *P*=0.0043 and at 100 ms interstimulus interval: 22.26±9.71% for sham versus 59.11±12.05% for THINs lesion; *P*=0.0357 unpaired *t*-tests).These results clearly implicate the THINs that are part of the disynaptic pathway leading to the thalamic inhibition of PLTS interneurons in the mediation of PPI.

## Discussion

Optogenetic activation of PfN thalamostriatal afferents evoked very different responses in the two populations of NPY-expressing striatal interneurons. While essentially all striatal NGF interneurons received a strong excitatory input from the PfN, the most common response in PLTS interneurons was a disynaptic inhibition. Our data demonstrate the existence of two parallel disynaptic thalamostriatal circuits, one disynaptic circuit comprises a late excitatory nicotinic EPSP in NGF interneurons triggered by monosynaptic PfN thalamostriatal glutamatergic activation of CINs. The other one leads to inhibition of most PLTS interneurons. The latter circuit involves thalamic glutamatergic excitation of striatal GABAergic interneurons comprised predominantly or exclusively of THINs that inhibit PLTS (Supplementary Fig. 7). We also provide evidence that this pathway is involved in the modulation of PPI of the startle reflex, an effect shown to involve a thalamostriatal pathway^40^,^42^,^43^,^48^.

Striatal NGF interneurons have only recently been recognized as a novel subtype of striatal GABAergic NPY interneurons^22^. In terms of extrinsic input, electrical stimulation of cortex evokes a monosynaptic EPSP in NGF interneurons that is smaller than in PLTS interneurons, and that is always subthreshold^22^. Here we showed nearly identical responses with optogenetic cortical stimulation. In sharp contrast, NGF interneurons receive a powerful excitatory input from the PfN that often triggers spiking.

NGF interneurons also receive powerful nicotinic input from CINs and exhibit a very high connection rate (>80%) with neighbouring SPNs, an exceedingly low failure rate, and an ususually slow, long duration IPSC (GABA_Aslow_)^19^,^22^. Given their consistent and powerful activation by the PfN, NGF interneurons are positioned to play an important role as mediators of a strong feedforward inhibition to SPNs from both PfN and CINs.

Approximately 25% of the NGF interneurons exhibited a second EPSP subsequent to the initial monosynaptic glutamatergic EPSP. This second EPSP was mediated by type II nicotinic, receptors. This is consistent with the recent description of a monosynaptic nicotinic excitation of NGF interneurons by CINs^19^, and with older reports of a predominant innervation of CINs by the intralaminar thalamic nuclei^12^,^13^,^15^. CINs responses that encode the sensory salience, reinforcement value and magnitude of expectation of external stimuli are dependent on normal thalamic activation^28^,^49^,^50^. Nevertheless, the dramatic contextual and learning-related flexibility of the CIN responses demonstrate that they are not directly controlled by the more stereotypical thalamic responses. Instead, the responses of CINs must reflect an integration of additional synaptic inputs from intra- and perhaps extra-striatal sources that provide the necessary contextual information for these interneurons^51^,^52^,^53^. We suggest that the variation of the responses of CINs are causally linked to a corresponding flexibility in the dynamic response of the entire striatal interneuronal circuitry. As a result, the phasic thalamic activation triggered by salient stimuli may be gated or modified by the dynamics of the circuitry that in turn reflects the animal's representation of the momentary behavioural significance of external stimuli.

The response of PLTS interneurons to optogenetic stimulation of PfN efferents was heterogeneous, with nearly one third of PLTS interneurons showing no response, consistent with rodent ultrastructural data^30^ The majority of the cells that did respond exhibited IPSP/Cs, with a small number exhibiting an excitatory response and a few cells receiving an overlapping mixed EPSP-IPSP sequence. Altogether, almost 80% of the PLTS interneurons in our sample failed to exhibit the expected monosynaptic excitation from PfN.

The onset latency of the PLTS IPSP/C was significantly longer than that of the SPN or NGF EPSP, consistent with its identification as a disynaptic response. THINs receive suprathreshold excitatory cortical input^21^ and here we show that the majority of them also receive a strong suprathreshold excitatory input from PfN. Furthermore, we showed that THINs provide efficient GABA_A_-mediated inhibitory input to PLTS interneurons. The amount of the suppression of the inhibition after optogenetic inactivation of THINs was variable. This variability may be attributable to (1) the position of the recorded interneurons with respect to both viral transduction fields in the striatum (for example, thalamostriatal fibres and most importantly their proximity to transfected THINs) and/or (2) the possibility that THINs are only partly responsible for the disynaptic inhibition of PLTS interneurons after optogenetic thalamic stimulation. However, the fact that for some cells the suppression of this inhibition is close to 75% suggests that THINs are likely the main, or perhaps the only, contributors to this inhibition.

*Ex vivo* whole-cell recording studies and biocytin fills show that there are four types of THINs, all of which have been shown to elicit IPSP/Cs in SPNs. However, the type I is by far the most frequently observed subtype, comprising between 66 and 80% of THINs identified in TH-EGFP and TH-Cre mice^21^,^54^,^55^ and in fact appears to be the only subtype reported in wild-type mice^56^. Further, in the present experiments, all of the THINs that were recorded were type I. Thus, although it is impossible at present to rule out a role for types II, III and VI THINs in the thalamostriatal disynaptic circuit and the modulation of PPI, it is certain that the type I THINS are involved.

We showed that ablating the THINs provoked a significant reduction in PPI of the acoustic startle response, without affecting the pattern, speed or overall distance travelled in an analysis of locomotor activity. Patients with obsessive-compulsive disorder, Tourette syndrome and Huntington's disease display clear deficits in PPI (see refs 48, 57, 58). Interestingly, one common substrate of those pathologies is abnormalities within striatal circuitry. Several lines of evidence suggest that the striatum may be one site of convergence between the neural disturbances in Tourette syndrome and the neural substrates of PPI^48^. Furthermore, deep brain stimulation of the PfN alleviates PPI^42^ and lesions of the striatum can disrupt PPI^43^,^44^ highlighting the probable role of the thalamostriatal pathway in this sensorimotor gating behaviour. Interestingly, a recent study show that ablation of CIN in the dorsolateral striatum, using the same method, reproduces some features of Tourette syndrome without replicating the deficit in PPI^59^. This highlights the distinct functions of the two parallel thalamostriatal pathways in this sensorimotor gating task. To our knowledge, these results are the first to show a specific role of the THINs in behaviour.

As previously demonstrated, THINs also provide inhibition to SPNs^22^. Even though this inhibition is relatively weak in comparison to the one provided to the PLTS interneurons, we cannot rule out that the loss of this inhibition is responsible, in part for the behavioural effect observed.

The striatal interneuronal circuitry is far more complex than originally anticipated, where striatal GABAergic interneurons were generally thought of as being basically the same (and often simply represented by the PV fast-spiking interneuron) that provided feedforward inhibition to SPNs and that are innervated by extrinsic glutamatergic inputs from cortex and thalamus, the latter often lumped together in conceptually and/or in diagrams.

However, the data presented here reveal a markedly more complex thalamostriatal and intrastriatal circuitry than previously envisaged. The differences between striatal PLTS and NGF NPY interneurons are much greater than their similarity. These two populations not only differ in their morphology, electrophysiological properties and protein expression, but also in their responses to extrinsic inputs from the thalamus and cortex and their intrastriatal connectivity. Our data show that there is great specificity between cortical and thalamic glutamatergic inputs to different population of GABAergic interneurons. These data, together with previous reports^19^,^22^ strongly suggest that the two subtypes of striatal NPY interneurons play very distinct roles in intrastriatal circuitry and behaviour.

Contrary to the traditional feed-forward model, our findings show that the impact of the PfN on the activity of the striatum depends critically on synaptic interactions within the interneuronal circuitry. This is directly demonstrated by the disynaptic inversion of thalamic excitation into an inhibition of PLTS neurons. More broadly, we suggest that the functional effect of thalamic inputs to the striatum may depend on the described complex and organized connectivity of the different types of striatal interneurons.

## Methods

### Animals

Subjects were adult (male and female, 3–6 months of age) bacterial artificial chromosome (BAC) transgenic mice that express the humanized *Renilla* green fluorescent protein (hrGFP) (Stratagene) under the control of the mouse NPY promoter (stock 006417; The Jackson Laboratory). To study the roles of THINs and their involvement in the disynaptic inhibition of PLTS interneurons, we used BAC transgenic TH–Cre [Tg(TH–Cre)12Gsat; Gene Expression Nervous System Atlas (GENSAT) as well as double transgenic mice TH-Cre x NPY GFP.

Offspring were genotyped and those found to be heterozygous positive for the transgene of interest were used for all recordings. All procedures were performed with the approval of the Rutgers University Institutional Animal Care and Use Committee and in accordance with the NIH Guide to the Care and Use of Laboratory Animals.

### Imaging

Mice were deeply anaesthetized with ketamine (100 mg kg^−1^). Brain tissue was fixed by transcardial perfusion of 10 ml of ice-cold artificial cerebrospinal fluid (adjusted to 7.2–7.4 pH), followed by perfusion of 90–100 ml of 4% paraformaldehyde, 15% picric acid in phosphate buffer. Fixed brains were extracted and post fixed overnight in the same fixative solution. Fifty to sixty micrometre sections were cut on a Vibratome 3000. Sections were mounted in Vectashield (Vector Labs, Burlingame, CA) and representative photomicrographs were taken using a confocal microscope (Fluoview FV1000, Olympus). Photomicrographs were taken using × 10 and × 60 objectives. Comparable pictures were taken using the same laser settings.

### Intracerebral viral injection

Mice were anaesthetized with isofluorane (1.5–2.5%, delivered with O2, 1 l min^−1^) and placed within a stereotaxic frame. Mice were injected in the thalamus or motor cortex with non-competent Adeno-associated virus AAV5-CAMKIIa-hChR2(H134R)-EYFP, or AAV5-CAMKIIa-hChR2(H134R)-mCherry (University of North Carolina, Vector Core Services, Chapel Hill, NC). To study the thalamic input to THINs, in addition to those virus injections in the thalamus, an AAV5-Flex Td Tomato virus (University of North Carolina, Vector Core Services, Chapel Hill, NC) was injected into the striatum of TH-Cre mice. To examine the role of those THINs in the disynaptic inhibitory responses in NPY-PLTS interneurons AAV5 Ef1a DIO HR3.0-mCherry (Penncore) was injected in the striatum of TH Cre × NPY GFP mice in addition to AAV5-CAMKIIa-hChR2(H134R)-EYFP injection in the thalamus. The surgery and viral injection took place inside a Biosafety Level-2 isolation hood. Bupivacaine was used as a local anaesthetic in the site of the surgery. Coordinates to target the CM/Pf of the thalamus were −2.3 mm anterior and 0.75 mm lateral to Bregma. A volume of 0.5 μl of virus was delivered by glass pipette to two sites −3.2 and −3.45 mm ventral to the brain surface. Coordinates to target the striatum were 0.74 mm anterior and 1.8 mm lateral to Bregma. Virus was delivered by glass pipette to three sites −2.25, −2.65 and −3.2 mm ventral to brain surface for a total volume of 1.2 μl. To target the cortex virus was injected to three sites that were 0.98 mm anterior 1.05, 1.55 and 2.05 mm lateral to bregma and 0.75 mm ventral to brain surface for a total volume of 1 μl. Virus was injected at 0.92 nl/5s, after which the pipette was left in place for 10 min before being slowly retracted. Expression of viral transgenes were allowed for at least 4–6 weeks before animals were used for histology or physiology.

### Preparation of brain slices

Mice aged 3–6 months were deeply anaesthetized with 100 mg kg^−1^ ketamine transcardially perfused with ice cold *N*-methyl D-glucamine (NMDG)-based solution containing (in mM): 103.0 NMDG, 2.5 KCl, 1.2 NaH_2_PO_4_, 30.0 NaHCO_3_, 20.0 HEPES, 25.0 glucose, 101.0 HCl, 10.0 MgSO_4_, 2.0 Thiourea, 3.0 sodium pyruvate, 12.0 *N*-acetyl cysteine, 0.5 CaCl_2_ (saturated with 95% O_2_ and 5% CO_2_, pH 7.2–7.4). After decapitation, the brain was quickly removed into a beaker containing the ice-cold oxygenated NMDG-based solution before obtaining 300 μm parahorizontal slices.

Oblique parahorizontal sections, 300 μm in thickness, were cut in the same medium using a Vibratome 3000. Sections were immediately transferred to recover in well oxygenated NMDG-based solution at 35 °C for 5 min, after which they were transferred to well-oxygenated normal Ringer's solution at 25 °C until placed in the recording chamber constantly perfused (2–4 ml min^−1^) with oxygenated Ringer's solution at 32–34 °C. Drugs were applied in the perfusion medium and were dissolved freshly in Ringer's solution.

### *Ex vivo* recordings

Slices were initially visualized under epifluorescence illumination with a high-sensitivity digital frame transfer camera (Cooke SensiCam) mounted on an Olympus BX50-WI epifluorescence microscope with a × 40 long working distance water-immersion lens. Once a GFP+ interneuron was identified, visualization was switched to infrared–differential interference contrast microscopy for the actual patching of the neuron. Micropipettes for whole-cell recording were constructed from 1.2 mm outer diameter borosilicate pipettes on a Narishige PP-83 vertical puller. The standard internal solution for whole-cell current-clamp recording was as follows (in mM): 130 K-gluconate, 10 KCl, 2 MgCl_2_, 10 HEPES, 4 Na_2_ATP, 0.4 Na_2_GTP, pH 7.3. These pipettes had a DC impedance of 3–5 MΩ. Membrane currents and potentials were recorded using an Axoclamp 700B amplifier (Molecular Devices). Recordings were digitized at 20–40 kHz with a CED Micro 1401 Mk II and a PC running Signal, version 5 (Cambridge Electronic Design). Optogenetic stimulation *in vitro* consisted of 2–5 ms duration blue light pulses delivered using a high-power (750 mW) LED. Sweeps were run at 20 s intervals.

### Drugs

Drugs that were used include bicuculline (10 μM, Sigma) to block GABA_A_ receptors, Dihydro-b-erythroidine hydrobromide (DhβE; 1 μM, Tocris) to block type 2 nicotinic receptors, methyllycaconitine citrate (MLA; 500 nM, Tocris) as an antagonist of type I nicotinic receptors, mecamylamine hydrochloride (MEC; 5 μm, Tocris), CNQX (10 μM, Tocris) and APV (10 μM, Tocris) to block, respectively, AMPA and NMDA receptors.

### THINS role in locomotor activity and acoustic startle reflex

For examining the effect of eliminating the THINs on habituation and PPI of the acoustic startle response, we used TH-Cre mice in which we injected an adeno-associated virus containing the neurotoxin DT-A (AAV5-EF1a-DIO-DT-A-mCherry, UNC vector core^60^) to destroy striatal THINS. Coordinates: AP:0.5 mm, ML:1.8 mm, DV:2.75 mm (330 nl) and 3.3 mm (440 nl) and AP:1.3 mm, ML: 1.0 mm, DV: 3.9 (160 nl). The Sham group consisted of TH-Cre mice in which we injected an adeno-associated virus containing the fluorescent protein mCherry (AAV5-EF1a-DIO-mCherry or AAV5-hSyn-DIO-hM4Di-mcherry, UNC vector core).

For open-field activity, all mice were habituated to a 40 by 40 cm open field chamber for 30 min. Twenty-four hours after habituation mice underwent open-field testing for 10 min. Spontaneous locomotor activity was recorded and analysed using Noldus Ethovision XT 11.5 software. The parameters measured were total distance travelled over 10 min (in cm) and average speed (in cm s^−1^).

The behavioural apparatus used for assessing habituation and PPI of the acoustic startle reflex was designed based on ref. 61 and constructed in house. Briefly, mice were restricted inside a Plexiglas cylinder (9 × 5 cm) to prevent movements that would interfere with the startle response. This cylinder was supported by foam plastic feet placed at the front and the rear. A piezoelectric-sensor was placed with a light contact underneath the cylinder. Two piezoelectric transducers (3.5 kHz, Mallory, Mouser MSR516NR) were used. One was placed on top of the cylinder (speaker 1) and the other one 5 inches away (speaker 2). The full apparatus was placed inside a soundproof isolated chamber. Sound was calibrated using a GoerTek sound level meter model TKsound). First, on an initial subset of mice an input/output function was measured to ensure that appropriate dB levels were used for the prepulse and startle stimuli. After an acclimatization period of 5–10 min with a constant background white noise of 60 dB, acoustic stimuli (20 ms) were displayed every 20 s, starting at around 65 dB. Acoustic stimulus intensity was increased between each stimulus by 2 dB. This allowed us to determine the startle threshold and use lower-intensity stimuli for the prepulse and higher-intensity stimuli for evoking startle. After these parameters were chosen, all mice underwent the full startle protocol that consisted of two blocks; a habituation block and a PPI block^62^. After a 10 min acclimatization period where no startle stimulus was displayed, mice underwent habituation in which 30 stimuli (20 ms, 100 dB) were applied every 20 s with speaker 1. For the PPI block, two parameters were tested: the interstimulus interval between the prepulse (4 ms duration) and the startle pulse and also the intensity of the prepulse. We used two different prepulse intensities (70 and 80 dB) and two interstimulus intervals (30 and 100 ms). Thus there are four different prepulse-startle trials plus the startle pulse alone trial and the prepulse alone trial, which make a total of six different trials. Each trial type occurred a total of ten times throughout the full session in a pseudorandomized manner. Inter trial interval was set to 20 s.

### Analysis

To measure the effects of ablation of THINs on the acoustic startle response, the startle responses of block I were plotted for each animal. Data from each animal were normalized to the maximal startle response of the three first trials, (animals sometimes fell asleep during the acclimation phase resulting in a low first or two firsts startle response and a higher second or third startle). The normalized data were then averaged across all animals. For a quantitative assessment of the amount of habituation, a score was calculated for each animal, for example, the average of the last five startle responses divided by the maximal startle response of the three first trials.

For PPI analysis, data were sorted by trial type, the integral of the 250 ms following the startle stimuli for each trial was measured and then averaged (Igor). The average value of the startle response for each condition was expressed as a percentage of the response evoked by the startle stimulus alone. This percentage revealed the amount of PPI induced by the particular prepulse condition.

### Statistics

All data are presented as the mean±s.e.m. Comparisons were made using Student's *t*-test or one-way ANOVA followed by Tukey's *post hoc* test.

### Data availability

All relevant data are available from the authors.

## Additional information

**How to cite this article:** Assous, M. *et al*. Differential processing of thalamic information via distinct striatal interneuron circuits. *Nat. Commun.* **8,** 15860 doi: 10.1038/ncomms15860 (2017).

**Publisher's note:** Springer Nature remains neutral with regard to jurisdictional claims in published maps and institutional affiliations.

## Supplementary Material

Supplementary Information Supplementary Figures

Peer Review File
