# Supplementary material for: Differential processing of thalamic information via distinct striatal interneuron circuits
Source: Nat Commun. 2017 Jun 12;8:15860. doi: 10.1038/ncomms15860 (PMC5477498; doi:10.1038/ncomms15860)
Supplement: Supplementary Information — Supplementary Figures [file ncomms15860-s1.pdf]

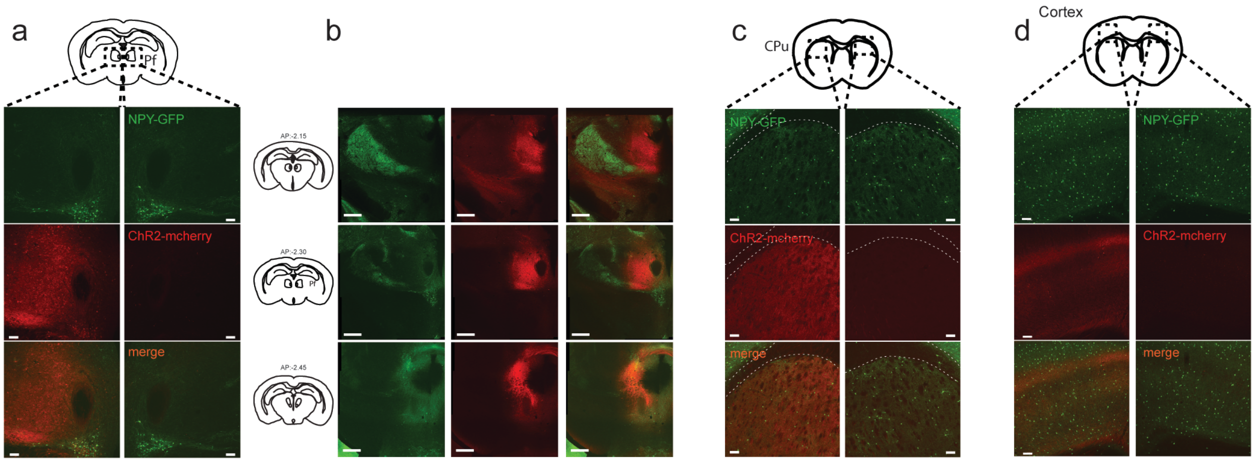

Supplementary Figure 1. Virus transfection in thalamus, striatum and cortex

(**a-b**) Confocal photomicrographs showing the parafascicular nucleus of the thalamus (Pf) of an NPY-GFP mouse showing the AV5-CAMKII-ChR2-mCherry transduction field around the injection site. (**a**) Left panels are the injected PfN, right panels are the non-injected side. Scale bar: 100 µm (**b**) Larger view of the transfection in the thalamic PfN in three antero-posterior levels (AP:-2.5; -2.30; -2.45 vs. bregma). Scale bar: 400 µm (**c**) Confocal photomicrographs showing the thalamostriatal axons in the striatum in red and the NPY-expressing interneurons in green. Scale bar: 100 µm (**d**) Confocal images of a cortical area showing that viral injection of virus in the PfN does not induce significant transduction of cortical neurons**.** Scale bar: 100 µm

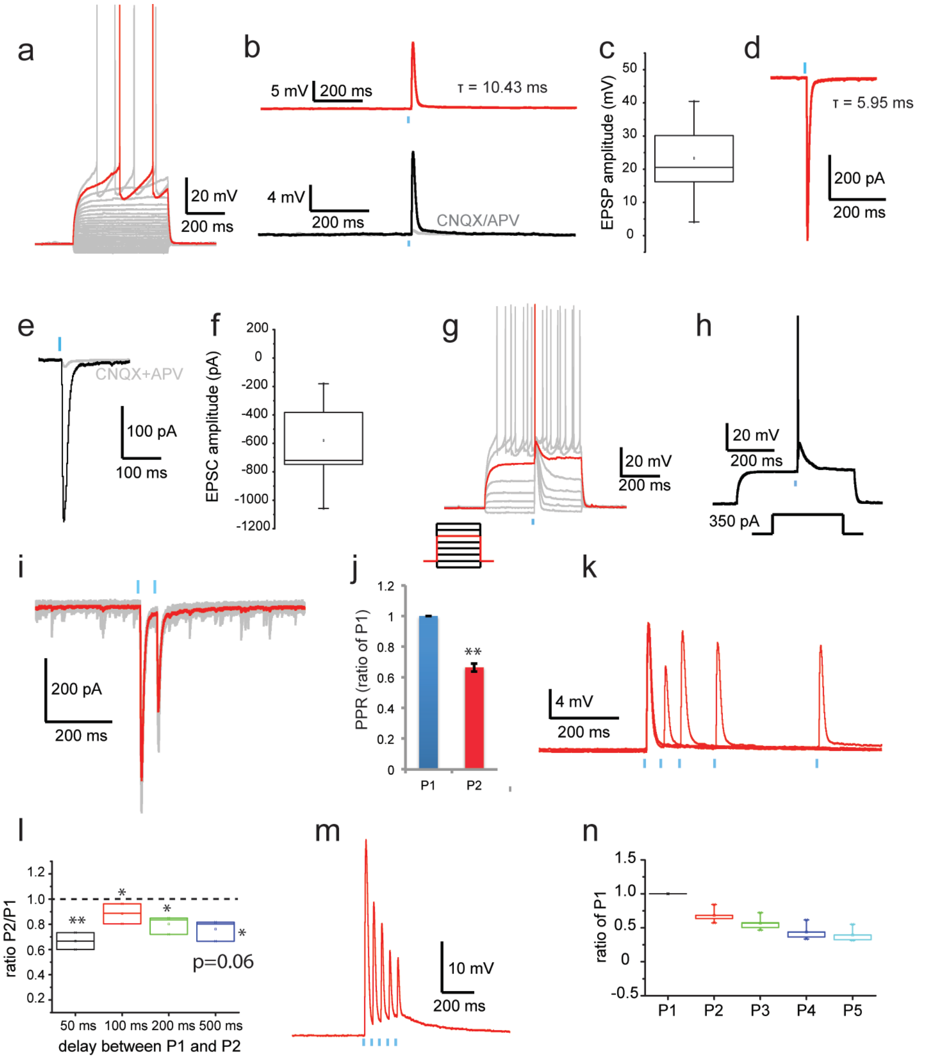

Supplementary Figure 2. Optogenetic thalamic stimulation provokes a strong depolarization of SPN.

(**a**) Responses of a typical SPN to injected current pulses. (**b**) EPSP in a SPN following optogenetic stimulation of PfN terminals. The large EPSP is almost completely abolished by bath application of 10 µM CNQX and 10 µM APV (lower panel, grey trace). (**c**) Box plot of the amplitudes of the EPSP provoked by the light stimulation (n=8)_. (**d**) Voltage clamp recording of a SPN following optogenetic stimulation of PfN terminals. (**e**) The large EPSC provoked by the light stimulation is dramatically reduced by bath application of a CNQX, 10 µM and APV, 10 µM. (**f**) Box plot of the amplitude of the optogenetic EPSC (n=7). (**g**) Responses of a typical SPN to injected current pulses and optogenetic stimulation. Note that the rheobase current is markedly reduced (**h**). (**i**) Paired pulse ratio (PPR) in a SPN after 2 brief optogenetic light pulses (inter pulse interval: 50 ms). Individual traces are shown in grey and the average in red. (**j**) Summary graph of the PPR data (n=4). (**k**) EPSPs elicited by paired optogenetic stimuli with increasing ISIs. EPSPs were recorded from the same cell. (**l**) Summary graph of PPRs recorded from SPNs plotted against interstimulus interval (n=4). (**m**) EPSPs elicited in a SPN after an optogenetic stimulus train (5 pulses, 20Hz). (**n**) Summary graph representing the ratio of the amplitude of the subsequent pulses divided by the amplitude of the first one (n=5). Bar graphs represent mean ± SEM. Box plots represent the interquartile range, the mean and median. * $p<0.05$, ** $p<0.01$ paired student t test (G), One way ANOVA (L).

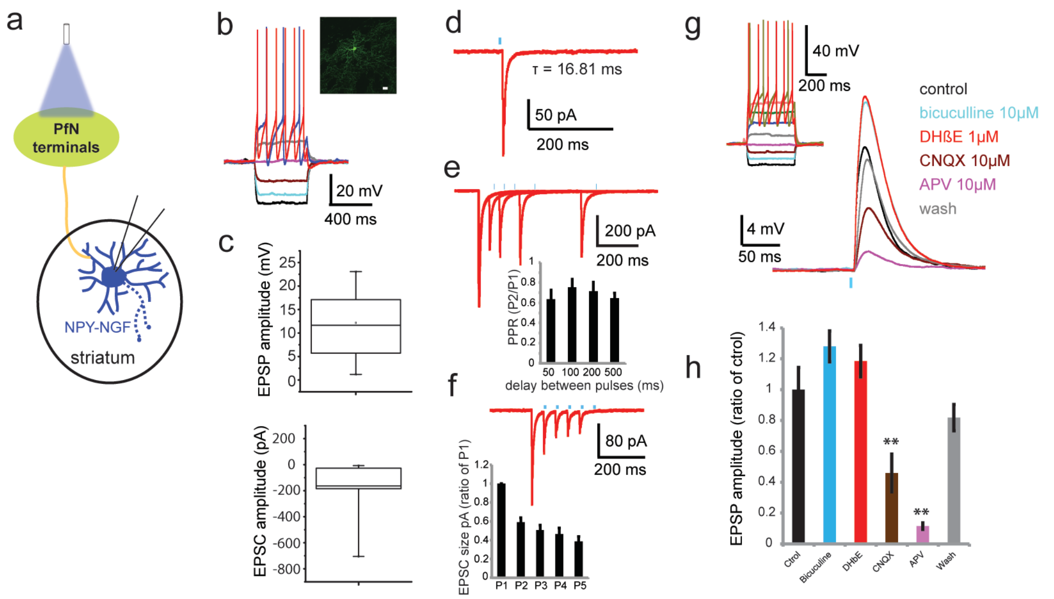

Supplementary Figure 3. Detailed characterization of the thalamic input to NPY-NGF interneurons (**a**) Schematic illustrating the experimental paradigm. (**b**) Responses of a typical NPY-NGF interneuron to injected current pulses. Note the very large spike AHP. (**c, g**). Summary graph representing the average EPSP/C amplitude elicited by optogenetic thalamic stimulation (n=12 and n=14 respectively). (**d**) NGF interneuron following optogenetic stimulation of thalamus showing a large EPSC. (**e**) EPSPs elicited by paired optogenetic stimuli with increasing ISIs. EPSPs were recorded from the same cell and summary graph of PPRs recorded from NPY-NGF interneurons plotted against interstimulus interval. (**f**) EPSCs elicited in NPY-NGF interneurons after an optogenetic stimulus train (5 pulses, 20Hz) and summary graph representing the ratio of the amplitude of the subsequent pulses divided by the amplitude of the first one (n=7). Bar graphs represent mean ± SEM. Box plots represent the interquartile range, the mean and median. (**g**) Representative traces obtained in current clamp showing the EPSP induced by optogenetic stimulation of the PfN and its modification after bath application of bicuculline, DHßE, CNQX, APV. (**h**) Summary graph showing that application of bicuculline provokes a slight non-significant increase of the thalamic-evoked EPSP, DHßE does not affect the size of the EPSP, whereas CNQX and APV dramatically reduce the EPSP size (n=7). Bar graphs represent mean ± SEM. ** $p<0.01$, one way ANOVA.

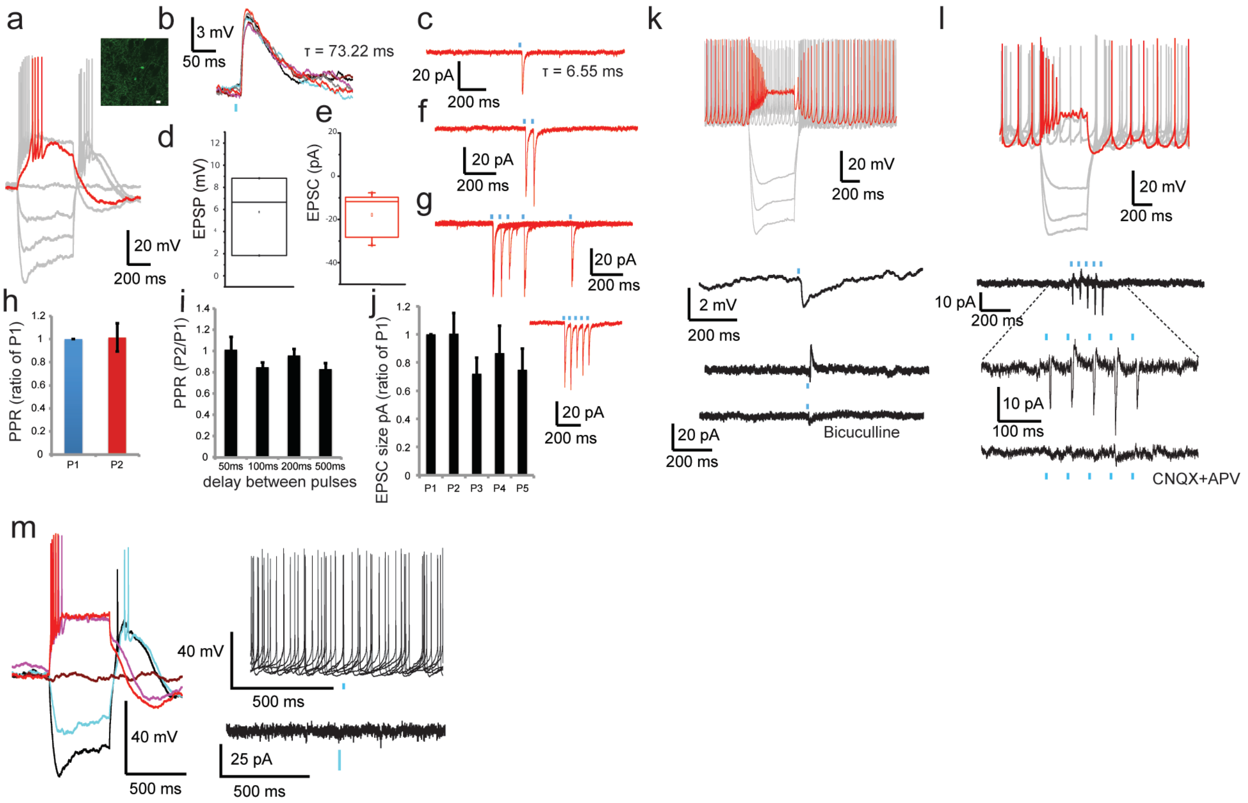

Supplementary Figure 4. Different types of responses evoked in PLTS interneurons after thalamic stimulation.

(**a, k, l, m**) Responses of typical PLTS interneurons to injected current pulses. Scale bar: 10 µm. Note the rebound spiking after offset of hyperpolarizing pulses and the depolarization block after large positive current injection Most neurons were spontaneously active (k, i). (**b**) EPSPs in a PLTS interneuron following optogenetic stimulation of PfN terminals. (**c,f,g**) EPSCs in a PLTS interneuron following optogenetic stimulation of PfN terminals.. (**d, e**) Box plots of the amplitudes of the optogenetic EPSPs and EPSCs (respectively n=4 and n=9). (**f**) Paired pulse ratio (PPR) in a PLTS interneuron after 2 brief optogenetic light pulse (inter stimuli interval (ISI): 50 ms). (**g**) EPSPs elicited by paired optogenetic stimuli with increasing ISIs. (**h**) Summary graph of the PPR data (n=5). (**i**) Summary graph of PPRs recorded from PLTS interneuron plotted against interstimulus interval (n=4). (**j**) Inset: EPSPs elicited in PLTS interneurons after an optogenetic stimulus train (20Hz) and summary graph representing the ratio of the amplitude of the subsequent pulses divided by the amplitude of the first one (n=5). Bar graphs represent mean ± SEM. Box plots represent the interquartile range, the mean and median. (**k**) Optogenetic stimulation of PfN terminals elicits IPSP in a PLTS interneuron. Lower panel: a PLTS following optogenetic stimulation of thalamus showing an early EPSC followed by an IPSC. Application of 10 µM bicuculline selectively blocks the IPSC leaving intact the EPSC. (**l**) Mixed excitatory/inhibitory response measured in voltage clamp (Vh = -45 mV) after optogenetic train stimulation of the thalamus in the same PLTS interneuron. Lower panel: expanded view of this mixed response showing that the excitatory component of the response is more precocious that the inhibitory one. Both components can be abolished by bath application 10 µM CNQX and 10 µM APV. (**m**) Example of a PLTS interneuron that was not affected by optogenetic thalamic stimulation (upper panel current clamp recording, lower panel voltage clamp recording).

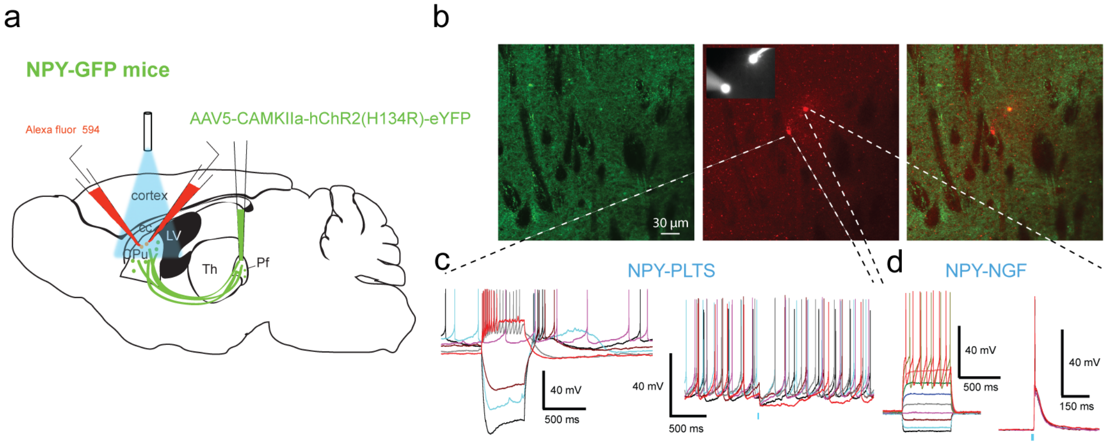

Supplementary Figure 5. Paired-recording of neighboring PLTS and NGF interneurons.
(**a**) Schematic illustrating the experimental paradigm where CAMKII-ChR2 virus has been injected in the thalamic PfN. Response of 2 neighboring PLTS and NGF interneurons were recorded and filled with Alexa Fluor 594. (**b**) Confocal pictures illustrating the recorded the NPY-GFP fluorescence (green), the recorded PLTS and NGF interneurons in red (and in white in the inset showing the 2 recording pipettes). (**c**) Responses of a typical NPY-PLTS interneuron to injected current pulses (left). Note that this PLTS interneuron respond to optogenetic thalamic stimulation by an inhibition (pause in the firing). (**d**) Responses of a typical NPY-NGF interneuron to injected current pulses (left). Note that this NGF interneuron respond to optogenetic thalamic stimulation by a very strong suprathreshold depolarization.

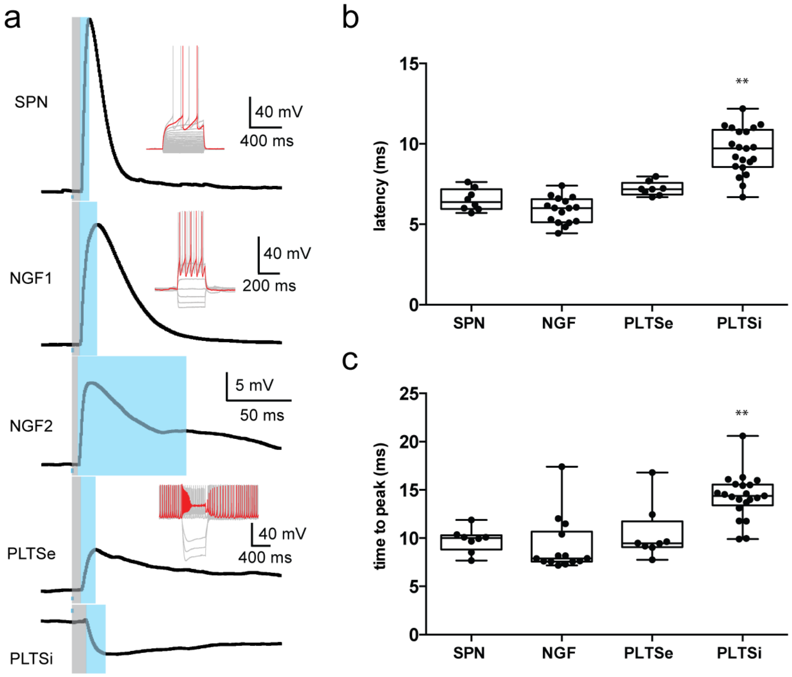

Supplementary Figure 6. Latency and time to peak of the different cell types responses after optogenetic thalamic stimulation.

(**a**) Responses to optogenetic stimulation of PfN terminals of the different cell types recorded. Grey and blue bars represent where we measured the values for the latency and the time to peak of the response. (**b**) Summary box-plots representing the latencies (n=8 SPNs, n=16 NGFs, n=8 PLTSe and n=21 PLTSi). Note the increased latency for the IPSP in PLTS interneurons consistent with a disynaptic response. (**c**) Summary box-plots of the time to peak (n=8 SPNs, n=16 NGFs, n=8 PLTSe, n=21 PLTSi). Box plots represents minimum, maximum, interquartile range, median and mean. * $p<0.001$, one way ANOVA followed by Tukey post hoc test.

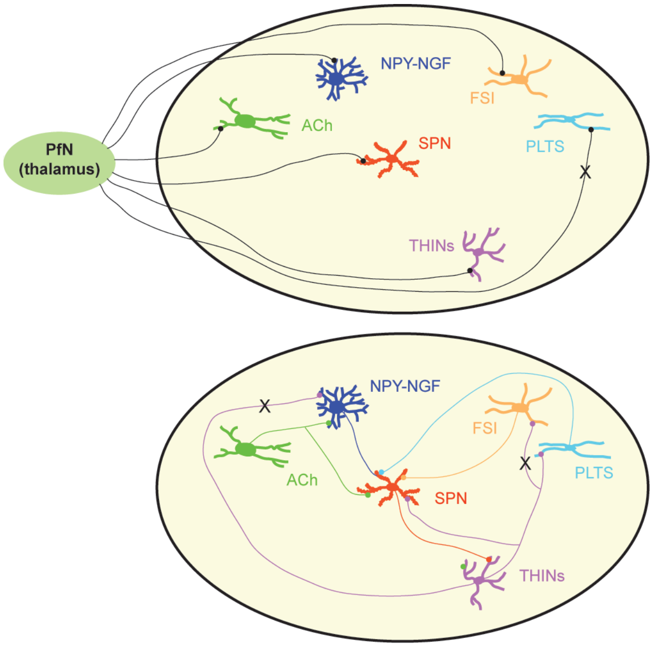

Supplementary Figure 7. Schematic summary of parafascicular nucleus innervation of striatal neurons.
Upper panel represents extrinsic thalamostriatal inputs to SPNs and interneurons. Lower panel represents intrastriatal synaptic interneuronal connections. X indicates a connection that was tested and found to be either weak or nonexistent.
