# Supplementary material for: Differential processing of thalamic information via distinct striatal interneuron circuits
Source: Nat Commun. 2017 Jun 12;8:15860. doi: 10.1038/ncomms15860 (PMC5477498; doi:10.1038/ncomms15860)
Supplement: Peer Review File [file ncomms15860-s2.pdf]

Editorial Note: this manuscript has been previously reviewed at another journal that is not operating a transparent peer review scheme. This document only contains reviewer comments and rebuttal letters for versions considered at Nature Communications.
